# The motives and methods of methamphetamine and ‘heroin’ co-use in West Virginia

**DOI:** 10.1186/s12954-023-00816-8

**Published:** 2023-07-12

**Authors:** Jeff Ondocsin, Nicole Holm, Sarah G. Mars, Daniel Ciccarone

**Affiliations:** Department of Family and Community Medicine, University of California, 500 Parnassus Avenue, MU3E, San Francisco, CA 94143 USA

**Keywords:** Opioid, Methamphetamine, Substance-related disorders, Polysubstance use, Ethnography, Injection

## Abstract

**Background:**

Opioid and methamphetamine co-use is increasing across the USA with overdoses involving these drugs also rising. West Virginia (WV) has led the US in opioid overdose death rates since at least 2013 and rising co-use of methamphetamine with opioids has played a greater role in deaths over the last 5 years.

**Methods:**

This study used rapid ethnography to examine methods and motivations behind opioids and methamphetamine co-use from the viewpoint of their consumers. Participants (*n* = 30) were people who injected heroin/fentanyl also using methamphetamine who participated in semi-structured interviews.

**Results:**

We found multiple methods of co-using opioids and methamphetamine, whether alternately or simultaneously and in varying order. Most prioritized opioids, with motives for using methamphetamine forming three thematic categories: ‘intrinsic use’, encompassing both inherent pleasure of combined use greater than using both drugs separately or for self-medication of particular conditions; ‘opioid assisting use’ in which methamphetamine helped people manage their existing heroin/fentanyl use; and ‘reluctant or indifferent use’ for social participation, reflecting methamphetamine’s low cost and easy availability.

**Conclusions:**

Methamphetamine serves multiple functions among people using opioids in WV. Beliefs persist that methamphetamine can play a role in preventing and reversing opioid overdose, including some arguments for sequential use being protective of overdose. ‘Reluctant’ uptake attests to methamphetamine’s social use and the influence of supply. The impact on overdose risk of the many varied co-use patterns needs further investigation.

## Introduction

Methamphetamine use among people who use heroin is increasing across the USA and is contributing to the ongoing overdose epidemic. The 2018 National Survey on Drug Use and Health (NSDUH) estimated that there were approximately 1.9 million past-year users of methamphetamine in the US, or 0.7% of the population, with 205,000 new initiates [1]. These numbers increased in both the 2019 and 2020 NSDUH, to 2.5 million past-year users by 2020, although in the 2020 NSDUH, new initiation of methamphetamine use fell to 150,000 [2, 3]. However, these counts may underestimate the extent of methamphetamine use due to disagreements between national surveys and other data sources which consider the extent of methamphetamine use to be much greater [4]. Other evidence finds that as access to prescription opioids was restricted, people who use opioids used methamphetamine in larger numbers [5]. Methamphetamine use among individuals seeking treatment for heroin dependence increased from 1 in 50 in 2008 to 1 in 12 by 2017 [6].

Since the 1970s accidental overdoses in the US have been rising exponentially albeit with significant regional, population and drug-type variability [7]. The last 20 years have represented three waves of opioid-related mortality with overdose deaths involving prescription opioids, heroin and synthetic opioids other than methadone (chiefly fentanyl) [8]. Adulteration of the US heroin supply is complicating this issue, with fentanyls largely sold as heroin or counterfeit pills driving the third wave of overdose deaths since 2013 [9–11]. Recent analyses suggest a fourth wave of deaths is already here attributable to both cocaine and psychostimulants—the latter class including methamphetamine—often in combination with opioids [12]. Fentanyl contamination of stimulants—which is likely accidental [10]—increased 2011–2016 [13] with significant regional variation. From 2013 to 2019, the age-adjusted death rate from psychostimulants, regardless of synthetic opioid co-involvement, increased 317% [14]. Illicitly manufactured fentanyls were co-involved in more than 80% of deaths involving stimulants over the first half of 2019 across 24 states [15].

The role of co-use of heroin and methamphetamine in overdose requires greater exploration. Intentional co-use of heroin and methamphetamine is increasing in the US, whether in simultaneous injection as a ‘goofball’, sequential injection or other combined modes, including smoking and snorting. Supply changes have played a part in widespread distribution of ‘ice’ or ‘cream’, a more potent and lower price Mexican-sourced methamphetamine supplanting the domestic product [10, 16].

‘Goofball’ was originally a term for barbiturate-type drugs with the earliest mention in the literature as heroin-methamphetamine injection in 2005 [17]. Heroin and methamphetamine co-use (referred to in some locations as a goofball and in others as a speedball, which historically has been a combination of heroin and cocaine) is spreading in locations as varied as Seattle, Washington; San Diego, California; Denver, Colorado; and Dayton, Ohio [18–21]. Qualitative research has found many people who use drugs (PWUD) believe methamphetamine can prevent or reverse opioid-related overdoses [20] and reduce withdrawal severity [22]. A study in Vancouver, Canada, found that practitioners used goofballs bi-directionally, both to enhance the individual effects of opioids and methamphetamine and to control for each drug’s negative effects [23]. However, knowledge about forms of co-use of methamphetamine and heroin, particularly from recent years, is needed to understand rising mortality among people using methamphetamine.

West Virginia (WV) has the highest rate of psychostimulant-involved deaths (24.4 per 100,000) of any US state [14]. To date little research has been conducted on the co-use of methamphetamine and opioids in this highly impacted community with most of the literature focusing on other US states and Canada. Local variations in patterns of drug use can be significant in shaping health risks [24]. Additionally, the elevated risk environment posed by co-use of powerful fentanyls and historically potent methamphetamine requires deeper investigation. A companion paper explores the role of drug supply changes in driving increased methamphetamine use among people who use opioids in West Virginia [25], while this manuscript focuses on how participants co-use these substances and the motivations behind such use.

## Background

West Virginia, part of the US Appalachian region, is largely rural, 93.1% white and has a 16.8% poverty rate, above the national rate (11.6%) [26]. Methamphetamine use has been endemic in WV since at least the 1990s, with the state serving as a distribution hub for the East Coast through the early 2000s [27]. WV overdose death rates have been among the highest in the country since at least 2013 [28]. Fatal overdoses related to methamphetamine and heroin/fentanyl co-use rose to half of all WV overdoses in 2018 [29]. Injection-transmitted HIV outbreaks have also been increasing in the state [30, 31].

## Methods

### Procedure

The Heroin in Transition study uses rapid ethnography to investigate emerging novel heroin forms and localities experiencing high levels of overdose [32]. The 'rapid ethnography' used in this project is a focused, short-term ethnography based on a methodology used to study disparities in HIV prevalence among urban populations in the 1990s [33]. Both methodologies center the observations and embodied experiences of participants, generating insider perspective and shared realities from a relatively underrepresented viewpoint. The rapidly changing nature of the drug supply and subsequent use patterns requires flexibility that traditional ethnography may lack, and provides an opportunity for comparison across multiple sites. Partnership with local agencies mitigated any challenges in recruitment and rapport-building, and unlike traditional ethnography, the short duration and lack of sustained presence provided greater anonymity for participants, allowing for more candid responses [32].

Following fieldwork in southwestern WV in 2017, we returned in 2019 to study the local uptake of methamphetamine among people using heroin/fentanyl. Five investigators carried out semi-structured interviews over a one-week period in September 2019. The interview guide covered topics including participants’ history of drug use, trajectory of drug injection, injection practices, methods of heroin and methamphetamine co-use, methods for determining drug adulteration, concerns about HIV, hepatitis C and other injection-related health consequences as well as overdose experience. Participants received $20 cash in compensation for their time (0.5–1 h). Ethnographic observations and photo/video recording of drug preparation and use, and tours of the locations and environments where participants lived, purchased and used drugs supplemented the interviews in fieldnotes written for each research day. Additional activities including photography of substance use and neighborhood tours were also compensated ($20). The University of California, San Francisco Committee on Human Research approved the study protocol and a Federal Certificate of Confidentiality protects the data and its collection.

### Recruitment and sample

Eligibility requirements included being at least 18 years of age, currently injecting ‘heroin’ and using methamphetamine through any means of administration. Exclusion criteria covered individuals who were intoxicated and unable to provide consent or answer questions. Individuals attending a syringe service program in southwestern West Virginia were approached, asked if they were interested in participating in the research study and provided formal oral consent. Some snowball sampling occurred. Interviews were audio recorded and all names given here are pseudonyms.

This was a non-random convenience sample with some purposive sampling to recruit women, often underrepresented in drug use research. Ethnographers conducted (*n* = 30) interviews with 12 cis-gender women and 18 cis-gender men aged between 24 to 49 years. Twenty-nine respondents self-identified as white or Caucasian and one as white and Native American. Experience injecting heroin ranged from 2 to 23 years while experience using methamphetamine ranged from 6 months to over thirty years. All but one participant initiated opioid injection before methamphetamine injection and one third (*n* = 10) initiated methamphetamine use in the 2 years before fieldwork.

### Analysis

Interviews were professionally transcribed and checked for accuracy by JO. JO, SM and NH wrote analytic memos for each individual transcript according to the methods used by Christopoulos et al. [34] and compiled them into a searchable table. Initial thematic categories were based on the structure of the interview guide, but later came to include co-use motivations, local drug market factors, effects associated with combined use in contrast to heroin or methamphetamine alone and a preliminary spectrum of co-use. Further analysis yielded distinct typologies and contrasting motives for co-use. Analysis prioritized how participants used and experienced heroin and methamphetamine, contextualized by the observations of the ethnographic team captured in fieldnotes that may have offered contrasting findings to what was reported by participants in interviews.

## Results

Motives for using methamphetamine with heroin/fentanyl can be conceptualized as forming three thematic categories: ‘intrinsic use’, representing the inherent pleasure of the combination or self-medication of particular conditions; ‘opioid assisting use’ in which methamphetamine helped manage existing heroin/fentanyl use and ‘reluctant or indifferent use’. All 30 individuals had some experience using methamphetamine, whether separately or combined with heroin.

We heard about and witnessed several ways that people used the two drugs, including simultaneous or alternating injections along a temporal spectrum. Daily order of dosing was another important and varied aspect of goofball use, with participants’ strategies dependent upon time of day, activity level, social situations and other factors. Most participants prioritized heroin over methamphetamine due to managing both opioid withdrawal and limited financial resources. Participants generally used the term ‘heroin’ to describe heroin, heroin adulterated with fentanyls and fentanyls without heroin; this language is reproduced in this paper to incorporate both drugs. Participants used the term ‘speedball’ to indicate co-injection of heroin and methamphetamine simultaneously, and generally did not use this term to refer to use separated in either time or mode of use (snorting, smoking). Ratios of heroin to methamphetamine within a speedball varied significantly among participants based on opioid tolerance, social situations and personal preference.

The co-use of heroin and methamphetamine, known locally as a ‘speedball’, a term used elsewhere to describe a cocktail of heroin and cocaine, gained popularity among our sample within the last several years, and was virtually unknown before approximately 2015. This was despite the earlier presence of domestically produced ‘shake and bake’^1^ methamphetamine in the local drug culture, albeit recently less widely available than the newer ice. Heroin was also a latecomer to the area, with evidence suggesting that the local market developed from 2012 onwards [35]. Among participants where the order of drug progression was clear, all had initiated their opioid use with prescription opioid pills, but had transitioned to heroin after the pills became prohibitively expensive and more difficult to obtain.

### Temporal spectrum of use

The ways in which these two drugs were combined or separated over time varied, although injecting as a mode of administration was universal. Some described a synergistic combination while others believed that using both substances together diminished their respective effects. Avery, in her 30s (using heroin < 10 years; methamphetamine a shorter time), preferred simultaneous use, favoring heroin and seeing ice as an enhancement but not a standalone drug:

*Q: So do you normally do speedballs like actual meth and heroin together or do you do them separately at different times of the day?*

*A: Oh no, I put that shit together.*

*[…]*

*Q: And if you only had a certain amount of money in a day would you choose one over the other?*

*A: Oh it would definitely be heroin. I mean because the only time I like the ice at all is to add […] to the speedball.*

Chase, in his 30s (using heroin 14 years; methamphetamine 4 years), found that a combined dose allowed him to feel both drugs sequentially which maximized the experience of using them together:

*Q: But when you do a speedball how it is different than if you’re just using them both back and forth?*

*A: You go up and down, I don’t know […] it makes it both the same. … if the heroin is good and the ice is good you’re doing the damn thing, you’ll be up and down, up and down.*

The duration of this effect or ‘legs’, Chase explained, depended on the quality of the heroin in particular:

*Q: And how long does it last that feeling?*

*A: It depends if it has legs or not. If it has good legs it should last for a couple hours.*

*Q: And what do you think gives something good legs?*

*A: How good the dope [heroin] is…*

Among individuals who preferred to inject heroin and methamphetamine separately, the time between each injection was highly variable. Paul, in his 20s (using heroin 6–7 years; methamphetamine 2–3 years) found that using the two in combination caused one to overpower the other, but did note some instances where he would inject a goofball:

*Q: Now do you use the heroin and meth separately or in combination?*

*A: Uh both, both from time to time. I feel like mixing them, one may draw from the other so like when you mix them you lose one of them. […] The only time I do them together is if I’m in like a hurry or only got a little bit of each.*

There was a high degree of variability in the order, timing and frequency of separate injections, with some preferring methamphetamine first in order to get ready for physical labor and remain active through the day, while others needed heroin first in order to allay withdrawal symptoms. Wyatt, in his late 20s (using heroin 6–7 years; methamphetamine < 6 years), preferred to use methamphetamine earlier in the day and save his heroin for nighttime: “Usually I’ll do meth first, where when I want to do the heroin is when I’m starting to get tired or sore or I want to chill, come down and watch TV or take a shower and relax and I’ll save that shot for relaxing.” Lisa, in her 30s (using heroin 6–7 years; methamphetamine < 6 years), described the long-lasting nature of methamphetamine relative to heroin, which she injected more frequently:

*Q: So what’s your typical day like drug-wise?*

*A: I wake up, I do a shot of dope.*

*Q: So you do just heroin in the morning?*

*A: Yeah, well it depends sometimes I do both but I make sure I have enough heroin that I won’t be sick if I do the meth. […]*

*Q: How long will the meth keep you high before you have to do that again?*

*A: Well I’ve been up 6 days high off of it.*

Allison, in her 20s (using heroin 5 years; methamphetamine 4 years), feared health consequences of simultaneous co-use, saying ‘I don’t like speedballing because it feels like my heart’s gonna explode.’ Instead, she chose heroin for pain relief on specific days but spaced the two drugs over time:

*Q: Okay. Tell me about a day where you choose to use heroin, why heroin that day?*

*A: Because my back’s hurting or I’ve got a really bad toothache or my legs are hurting and it takes the pain away.*

*Q: […] Or do you ever have a day like that where you use heroin at one point in the day and meth at another point in the day?*

*A: Yeah but as long as I use it, don’t use it in the same, if there’s at least 2 and a half hours within me using it that’s fine.*

### Motives for adding methamphetamine to opioid use

#### Intrinsic effects

For most participants, opioid initiation preceded the uptake of methamphetamine. Several sought out methamphetamine for its pleasurable effects, both alone and in combination with heroin. Alexander, in his 30s (using heroin 13 years; methamphetamine 2 years), attempted to explain what he called the “unexplainable” embodied experience of combined methamphetamine and heroin:

*You’re on top of the world. You know what I mean? You’re just, you feel good, you know you’ve got that warm, fuzzy feeling from the heroin and then you’ve got the euphoria feeling you get off the ice you know, just a mixture or a combination of both it’s kind of unexplainable.*

While some desired methamphetamine’s euphoria, a few participants implied that they used methamphetamine to self-medicate for various medical or psychiatric conditions. Owen, in his late 20s (using heroin and methamphetamine for unknown durations), discussed several conditions for which he found methamphetamine therapeutic:

*…Really how I know when I’ve done a good shit [sic] of ice is because I’ll yawn, get the munchies and ready to take a nap and that’s off the speed. But I’m also ADD, ADHD, ODC [sic], bipolar, split personality, explosive anger and very rambunctious. […] So I’m hyper as can be. I do a shot of speed and it slows me down…*

#### Opioid assisting

Many who felt neutral—or even negatively—about methamphetamine provided alternative reasoning or justification for its use. One emergent theme was that participants used methamphetamine as a tool to support their heroin use in various ways. From providing energy needed to acquire heroin, to preventing and reversing overdose, to managing aspects of dependency and withdrawal, participants found methamphetamine to be advantageous in their lives.

##### Energy supporting economic activity

For several participants, using methamphetamine supplied the energy needed to make the money to purchase heroin. Riley, in her 30s (using heroin 10 years; methamphetamine 6 months), self-described as “not really a speed person”, reported that methamphetamine supported her heroin use, allowing her to stay awake for days to sell methamphetamine, commenting “[…] wow, this stuff [methamphetamine] I gotta take this because hell, I got people that stay up for 5 days, I gotta stay up for 2 days at least to sell it, you know […] and I needed that to support my dope habit.”

##### Prevention and reversal of opioid overdose

In addition to providing energy, several participants believed that methamphetamine played a significant role in preventing overdose from both heroin and fentanyl. Harper, in her 30s (using heroin 13 years; methamphetamine for an unknown duration), said using heroin and methamphetamine together “keeps me from overdosing”. She explained “actually what’s saved my life, was the meth, you know over the heroin.”

Similarly, Alexander discussed the physiological effects of both drugs and expounded the theory behind his belief in methamphetamine’s overdose prevention mechanism:

*I like doing them both too, especially if it’s the fentanyl because it keeps my heart rate up as well as you know not ODing. […] fentanyl just slows your heart rate down and stuff like that—it just shuts you down. And the meth will keep you going. I know it’s bad on your heart and it’s gotta be but I do believe that it does has some type of protection of overdose, the meth does.*

In addition to overdose prevention, several participants believed in the ability of methamphetamine to reverse an opioid overdose. Oliver, in his 40s (using heroin and methamphetamine for 23 years) advanced methamphetamine as an alternative to “Narcan”, saying “I can give you a shot of meth instead of Narcan and it’ll bring you out of it…”, but conceded that it did not work all the time.

Cameron, in his 20s (using heroin and methamphetamine for unknown durations), was convinced of methamphetamine’s ability to reverse the effects of an opioid overdose, having witnessed it before::

*[…] I’ve seen it with my two eyes. They, I mean, they weren’t in a full-fledged – they weren’t dead, dead, but they were well on their way to going into a full-fledged overdose. […] I’ve been around plenty of people that overdosed and you know when they’re about to overdose. And somebody can – usually somebody else has to shoot ’em, but it gives ’em a large shot of meth, it’s like shooting with adrenaline will bring ’em back a little bit, enough to keep ’em alive, you know, so.*

However, he considered sequence of use to be critical in its effectiveness reporting that using methamphetamine before using heroin would diminish the protective effect:

*If I do a shot of heroin I can instantly do a shot of meth. […] But if I do a shot of meth, you’re gonna wanna wait a little while before you do heroin. I don’t know why, but that’s just the way it is. […] But, you know, if you’re on the ice already and you do heroin and you do too much, then it’s not gonna work the same.”*

Oliver, who has a respiratory condition, also preferred using methamphetamine after heroin: “The meth comes in later on in the evening after I’ve had a couple doses of heroin and I’m actually well and good and high. And then I want to do an extra activity.” Oliver noted that using methamphetamine prior to using heroin improved his breathing, allowing him to use larger amounts of heroin: “And of course it helps my lungs because believe it or not it helps me breathe. […]Yeah it kick-starts my lungs and like I can breathe. I can actually do a bigger shot of heroin after I do a shot of meth.” Others, however, preferred to use heroin after methamphetamine for personal or pleasure-based reasons, or did not identify a preference or benefit of different sequences of use.

Quinn, in her 20s (using heroin 11 years; methamphetamine 2–3 years) and former speedball consumer, represented a viewpoint held by some that simultaneous use was particularly risky for overdose but did not see any particular order as safer for preventing overdose: “But my cousin had died doing that [speedballing] so it scared me. So I quit you know, I quit doing that and the only thing I would do was I would do it separate. I would either do the heroin first and then the meth or the meth and then the heroin. I will not mix it together.”

### Managing opioid dependency

In general, participants prioritized obtaining heroin in order to stave off withdrawals, as Oliver explains:

*Well I usually I wake up dope sick, so I have to have the heroin. Ice gives you body aches and cramps and dehydrates you. So if you’re hydrated you’re fine but it’s not something that makes you sick like heroin does. […] So I have to have the heroin or I will get sick.*

However, due to the relative availability and price differential of methamphetamine as compared to heroin during the time of this study—an 8-ball (an eighth of an ounce) of methamphetamine cost from $40–80, while a tenth (one tenth of a gram) of heroin ranged from $10–20—some participants noted using methamphetamine to alleviate symptoms of withdrawal when they did not have heroin. Lucas, in his 30s (using heroin 5 years; methamphetamine < 5 years), recounted using methamphetamine to manage withdrawal:

*I was dope sick, I didn’t have any dope. […] I called my friend to help me and he said, all he had was some meth and that it would help me. And so I was like, okay, you know I was really sick. And when I shot it, it made the sickness go away. It did. I forgot completely about it.*

While multiple participants recounted similar experiences, Cameron said that using methamphetamine just delayed the onset of opioid withdrawal. Jackson, in his 40s (using heroin 20 years; methamphetamine 6 months), said methamphetamine use intensified opioid withdrawal: “…anytime I do methamphetamine or any type of speed, it really just makes me more dope sick from the heroin if I don’t have it.”

However, this viewpoint was more exceptional. More commonly, methamphetamine alleviated physical symptoms, but also provided a mental distraction that allowed participants to forget about heroin. Busy, focused activities such as filling in coloring books were common among people using methamphetamine.

*[…] it’s almost like [how] methamphetamine psychosis works, like I’m not even thinking about heroin. You’re thinking about coloring books and stuff like that, you know, you see what I’m saying.*

-- Cameron

*I was mixing in heroin. And doing the meth, ice it kind of backed me off the heroin. […] It focused me more into the meth you know it kind of made me, “Who needs that?”*

-- Alexander

Not trying to quit heroin altogether, Chase noted using methamphetamine to induce short-term opioid detoxes to reduce his tolerance:

*A: Sometimes I detox myself.*

*Q: Why do you do that?*

*A: So I can lower down my tolerance so I ain’t gotta do so much to get high.*

*Q: And how do you do that?*

*A: I detox myself off of heroin by using ice.*

A minority of participants did not seek out methamphetamine but did report circumstances in which they would use it.

#### Reluctant or indifferent use

Among those not using methamphetamine for its intrinsic or opiate assisting effects, there was a common theme that we have termed ‘reluctant’ or ‘indifferent’ use. During this time, methamphetamine was so pervasive and relatively inexpensive that several participants commented on its freely traded nature. Wyatt, did not seek out methamphetamine but used it if freely offered, “I might do a little meth once a week or once every other week or something but it’s mostly just extra if a friend offers….” Other participants also commented on using methamphetamine if freely given, despite their dislike of methamphetamine:

*I don’t like it. I do it often, I’ve never paid for it. I’ve never paid for it because ice especially around here is practically free. A week’s supply of ice costs about 40 bucks.*

-- Liam, in his 30s and using heroin 8 years; methamphetamine 5 years

Despite widespread experience using goofballs among participants, some, like Emma, in her 40s (using heroin 4 years; methamphetamine < 4 years), did not like the effects of methamphetamine and preferred to not use it at all, although she would use speedballs socially:

*Q: Is there anything that you – tell me what you don’t like about ice?*

*A: What I don’t like about it? I don’t like that it burns, I don’t like the buzz, I don’t like anything about it really, I just like the first 5 minutes.*

*Q: […] And is that also true with the speedball combination of it?*

*A: I only do that if people is like doing it, making it for free.*

Like Emma, a social aspect influenced several participants’ use. Natalie, in her late 30s (using heroin 5 years; methamphetamine 2 years), disliked methamphetamine but would use it socially with her boyfriend and to synchronize their time together:

*Q: And what do you like about meth?*

*A: I don’t really like it. (Laughs)*

*Q: So when you choose to do it what’s the situation like that […] you’re using meth?*

*A: My boyfriend loves it. And I do it mainly to stay up with him.*

## Discussion

This study builds on the evolving polysubstance use literature by examining a wide range of patterns and motivations for combining methamphetamine and opioid use in a specific, high-risk locale. For those who preferred to use each drug separately, the timing was highly variable: some prioritized opioids to prevent withdrawal symptoms, while others sought out methamphetamine first for energy provision. Order of use was also seen as significant in the prevention or reversal of opioid overdose, although views were inconsistent, with some claiming that heroin before methamphetamine was the most protective and others advocating the opposite. Simultaneous speedball use was perceived by some as particularly risky.

Among those who used both substances concurrently, either by preference or reluctantly, some reported a synergistic effect, while others considered that one drug, usually methamphetamine, would overpower or cancel the effects of the other. A possible explanation of the popularity of the previously unusual mixture of heroin and methamphetamine is the presence of fentanyl; much more potent than heroin, fentanyl may be a more equipotent match for methamphetamine making a ‘super speedball’, but further data is needed.

Polysubstance use may increase the risk of opioid overdose [36, 37] but although studies have found associations between overdose and combined use of heroin with other sedatives [38], the literature on overdose risk from heroin-stimulant combinations is limited [19, 39]. Goofball use has been associated with larger networks of PWID [19], which while potentially protective against overdose, may increase the likelihood of sharing injection equipment and contribute to transmission of bloodborne infections. Additionally, participants reported using methamphetamine to alter their sleep schedules or that methamphetamine use kept them awake for days, and the impact of sleep disturbances on susceptibility to opioid overdose needs additional study.

Consistent with other studies [20], several respondents strongly believed in the ability of methamphetamine to prevent and reverse opioid overdose. Set against this are statistical data that show greater frequency of overdose among individuals co-using methamphetamine and heroin compared to people solely using heroin [19, 40] and rising numbers of deaths involving combinations of fentanyl and methamphetamine [41]. Increased mortality from co-use of these substances could represent greater co-use of methamphetamine overall or escalating fentanyl saturation of the opioid market but does not explain the lay belief in the possible protective effects of methamphetamine against opioid overdose.

A potential explanation arises from recent mouse-model data which shows a bi-directional effect of amphetamine on fentanyl-depressed respiration depending on amphetamine dosage. Lower amphetamine doses depressed respiration after fentanyl, increasing the likelihood of overdose but higher amphetamine doses elevated respiration [42]. If applicable to humans, this finding may help to explain the apparent contradiction of methamphetamine both increasing and reducing the risks of fatal overdose and could lead to the development of important harm reduction strategies. However, more specific research on this drug interaction in humans is needed to understand this causal pathway.

The importance of dosage and drug sequence when using methamphetamine to mitigate adverse respiratory effects of opioids, fentanyl in particular, requires further study. Order of use may not be evident in post-mortem toxicology and the interaction of these drug mechanisms over time needs further exploration. Amidst rising amphetamine-related hospitalizations [43, 44], research should also consider other specific morbidity risks posed by co-use of opioids and methamphetamine, including how non-injection modes of use (i.e. smoking, snorting [45]) may impact morbidity and mortality (e.g. by reducing HIV/HCV or overdose risks).

Findings are from an exploratory, qualitative study based on a small convenience sample. Recruitment from a public health setting may have produced institutional or social desirability biases. However, interviews took place where participants felt comfortable and the semi-structured format allowed for response validation. Ethnographic observation around drug preparation, use and patterns of daily lives allowed for triangulation with interview data.

Previously published work has not yet explored the reluctant or indifferent use of methamphetamine among people who use opioids that is highlighted here. Several participants, with agnostic or negative attitudes about methamphetamine, reported methamphetamine use in specific, usually social, scenarios. The role of supply in driving the uptake of methamphetamine use among people using opioids is another salient factor. Widespread availability and the low price point of methamphetamine, combined with increasing levels of social use created greater opportunities for exchange among different social networks, leading methamphetamine to be available to individuals who may not otherwise have sought it out. If desire to use methamphetamine, whether for intrinsic or opioid assisting purposes, is not driving some methamphetamine use, this may provide opportunities for interventions that disrupt the social reinforcement of use.

## Data Availability

Data and materials are of a sensitive nature and are consequently not available to those outside of the study team. A US Federal Certificate of Confidentiality issued by the National Institutes of Health/National Institute on Drug Abuse protect the data and its collection.

## Footnotes

1 The ‘shake and bake’ form of methamphetamine production is a simplified version of the birch method of methamphetamine production, popularized in the 2000s as a response to US efforts to make precursor chemicals more difficult to obtain. This method uses only one vessel, often a two-liter plastic bottle, and low quantities of precursor chemicals to produce methamphetamine. It is also an extremely adaptable method of producing methamphetamine, accommodating various amounts of included precursor chemicals, albeit dangerous.
